# Direct Reduction of Fe, Ni and Cr from Oxides of Waste Products Used in Briquettes for Slag Foaming in EAF

**DOI:** 10.3390/ma12203434

**Published:** 2019-10-21

**Authors:** Arkadiy Davydenko, Andrey Karasev, Björn Glaser, Pär Jönsson

**Affiliations:** Department of Materials Science and Engineering, KTH Royal Institute of Technology, SE-100 44 Stockholm, Sweden; arkadyd@kth.se (A.D.); bjoerng@kth.se (B.G.); parj@kth.se (P.J.)

**Keywords:** EAF, stainless steel, slag foaming, waste products, recycling, briquettes

## Abstract

Environmental aspects and the sustainable manufacturing of steels require producers to pay more and more attention to the efficient utilization of materials and waste products during steelmaking. This study is focused on the evaluation of possibilities for the recovery of metals (such as Fe, Ni and Cr) from waste products used for slag foaming in the Electric Arc Furnace (EAF) process. Two types of industrial briquettes were produced by mixing mill-scale from the hot rolling of stainless steels with anthracite and pet-coke, respectively. Thereafter, an assessment of the metal reduction processes in briquettes at high temperatures (1500 °C) was made by using laboratory thermo-gravimetric reduction experiments in an argon atmosphere. The amounts of metal, slag and gas obtained from the briquettes were estimated. In addition, the velocity and time for the removal of metal droplets from the liquid slag depending on the size of the metal droplets was estimated. It was found that up to 97% of metal droplets can be removed from the slag during the first 30 min. Moreover, results showed that most of the Cr, Ni and Fe (up to 93–100%) can be reduced from oxides of these metals in briquettes at 1500 °C. Moreover, the anthracite and pet-coke in the investigated briquettes have similar reduction capabilities. It was found that up to 330 kg of Fe, 28 kg of Ni and 66 kg of Cr per ton of added briquettes can be recovered from waste products by the industrial application of those briquettes for slag foaming in EAF.

## 1. Introduction

Today, the effective application of energy and recycling of waste products in the steel industry is becoming more and more important for the sustainable development of the steelmaking industry. The production of stainless steels includes many energy consuming stages such as scrap melting in the Electric Arc Furnace (EAF), melt decarburization in the Argon Oxygen Decarburizer (AOD), melt refining in a ladle, casting, rolling, etc. Moreover, many different types of wastes (such as mill-scale, dust, sludge, etc.) are formed during all these stages of steel production. For instance, an amount of 30 to 70 kg of mill-scale per ton of rolled products can be obtained just during a rolling operation, depending on the rolling process conditions [1]. This means that the amount of waste mill-scale from only the rolling process corresponds to on average 5% of the total production of stainless steels. According to the annual world production of stainless steels [2], on average 1.2–2.1 Mton/year of mill-scale is formed during the production of 25–42 Mton/year of stainless steels. Utilization of such residues is of significant interest to the steel industry in Sweden. According to the Swedish Steel Association Jernkontoret [3], residue products such as slag, sludge, dust and mill-scale generally are sold or reused internally, otherwise they are sent to landfills.

Currently, the EAF process is one of the most energy consuming process used for the production of stainless steel. A more effective utilization of electric energy can be achieved by using slag foaming in the EAF process. It is well known that the foamed slag in the EAF can significantly help to decrease heat loss, the wear of furnace refractory and dust emissions. Slag foaming during the EAF process can be obtained by the additional formation of CO and CO_2_ gas bubbles in the slag, created by the addition of different foaming agents such as waste metal oxides [4,5,6,7,8,9], CaCO_3_ [4,5], Ca(NO_3_)_2_(H_2_O)_2_ [5,10], CaC_2_ [11], NiO+C [5], FeSi [12], doping agents (Fe, C, Si, Mn, O_2_) [13,14] and MgCO_3_ [15]. The addition of foaming materials into the furnace slag is usually carried out by injection and/or by addition of special briquettes. In general, these briquettes contain both reducible oxides as well as a reducing agent such as carbon. This mixture of oxides and carbon should provide gas generation at the high temperatures (≥1500 **°**C) required for slag foaming to occur during the EAF process. According to thermodynamic considerations, such oxides containing iron, nickel and chromium can be reduced by carbon at temperatures of ≥1500 **°**C. The mill-scale from stainless steel production, which contains mostly a mixture of these oxides, can be used as the main component for manufacturing the foaming briquettes. Thus, the application of such briquettes can be an efficient way for simultaneously slag foaming in the EAF and recycling waste products obtained from the steelmaking industry.

Some researchers in recent years have demonstrated that it is possible to utilize such briquettes with mill-scale in BOF [16] and the EAF steelmaking process [17]. In addition, researchers have reported that it is possible to create slag foaming in the EAF by such briquettes [7,18]. Furthermore, Lopez et al. [19] and Yang et al. [20] have reported that it is possible to use recycled metal oxides from the briquettes to create slag foaming in the EAF. However, some aspects regarding the feasibility and kinetics of recovering valuable metals (Fe, Ni and Cr) from the briquettes with mill-scale during slag foaming in the EAF need to be studied more in detail.

The aim of this article is a laboratory investigation the possibilities of recovering Fe, Ni, and Cr oxides by different reducing agents in slag foaming briquettes. Different reduction agents were mixed with mill-scale into briquettes used for EAF slag foaming. Moreover, the briquettes’ capacity for slag foaming under ideal conditions was estimated with a view to trials under industrial conditions. Slag foaming under industrial conditions with these types of briquettes has been studied previously [21].

## 2. Materials and Methods

**Figure 1:**
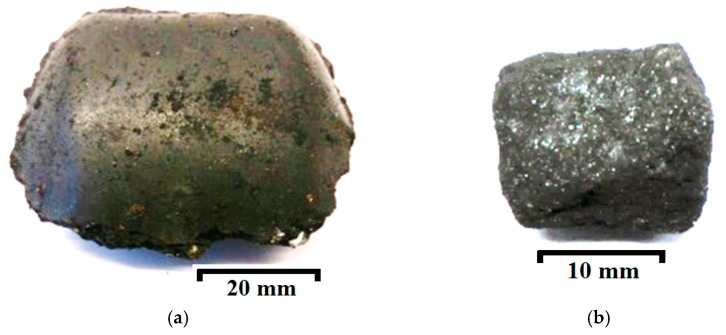
Typical images of an industrial briquette (**a**) and a briquette sample used in the thermo-gravimetric reduction (TGR) experiments (**b**).

Two types of briquettes, which can be used in the EAF to improve the foaming of the furnace slag, were produced on an industrial scale. The composition recipes for both types of briquettes have been described in a previous study [22]. Anthracite and pet-coke waste was used as a source of carbon in the Type A and Type C briquettes, respectively. Additional raw materials used for briquette production were lignin (~5%), slaked lime (Ca(OH)_2_, ~3%), limestone (CaCO_3_, ~5%) and mill-scale (70–72%) formed during the hot rolling of stainless steels. Here and below the amounts of components are given in weight percentage. The average chemical compositions of the obtained industrial briquettes are given in Table 1. The typical photographs of the obtained industrial briquettes and briquette samples used in the laboratory trials are shown in Figure 1. The briquettes were manufactured by crushing, raw materials screening, mixing and hot-pressing operations.

**Figure 2:**
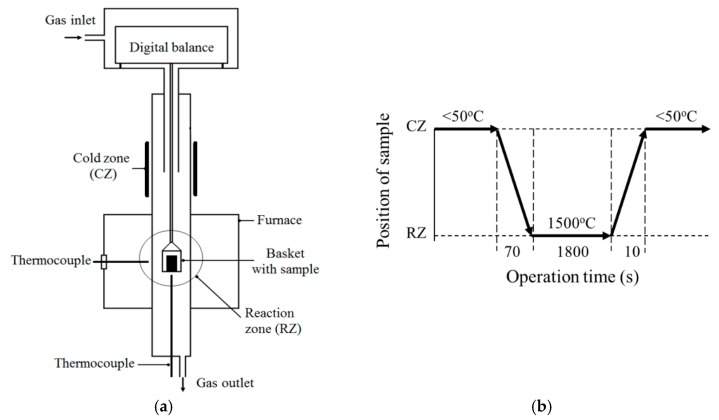
Schematic illustrations of the equipment setup (**a**) and the main operational description of the TGR experiment (**b**).

An evaluation of reduction processes in briquettes at high temperatures was made by using laboratory thermo-gravimetric reduction (TGR) experiments. Schematic illustrations of the equipment setup and main operations of the TGR experiments are shown in Figure 2. The TGR experiments were performed in a resistance furnace with a controlled argon gas (99.99% Ar) flow (0.1 L/min). The initial weight of the briquette samples (Figure 1b) was in the range of 7 to 8 g. After heating the reaction zone (RZ) of the furnace up to 1500 °C, an Al_2_O_3_ crucible with a briquette sample was lowered for 70 s from the cold zone (CZ) of the furnace (<50 °C) into the reaction zone (RZ). After 30 min of holding in the reaction zone, the basket with the crucible was lifted up to the cold zone of the furnace and kept there to cool the sample. The reduction of the sample weight due to gas generation during the experiment was measured continuously by using a digital balance. Moreover, after each TGR experiment, the metal and slag, which were formed during reduction processes in the briquette sample, were crushed, separated by using a strong magnet, and weighed. In total, three TGR experiments were carried out for each type of briquette using the same experimental conditions. Detailed descriptions of the performed TGR experiments are given in a previous article [22].

Samples taken before and after completion of the TGR experiments were dry polished and investigated by using a scanning electron microscope (SEM) equipped with an energy-dispersive spectroscopy (EDS) analyzer. The average compositions of the metal and slag phases were determined at least three times in different zones by using the area scan mode of EDS. 

The removal of formed metal droplets from the unstirred slag melt was evaluated by using the following version of Stokes law:

(1) $$\upsilon_{\left(\mathrm{MD}\right)}=\frac{2g}{9}\cdot \frac{\rho_{\left(\mathrm{MD}\right)}-\rho_{\left(\mathrm{slag}\right)}}{\eta_{\left(\mathrm{slag}\right)}}\cdot {\left(\frac{D_{\left(\mathrm{MD}\right)}}{2}\right)}^{2}$$

where υ_(MD)_ is the sinking velocity of the liquid metal droplet in the unstirred liquid slag having a Reynolds number <1; g is the gravitational acceleration (=9.81 m/s^2^); ρ_(MD)_ and ρ_(slag)_ are the densities of the metal droplets and slag melt. The parameter η_(slag)_ is the dynamic viscosity of the liquid slag and D_(MD)_ is the diameter of the formed metal droplets.

## 3. Results and Discussion

**Figure 3:**
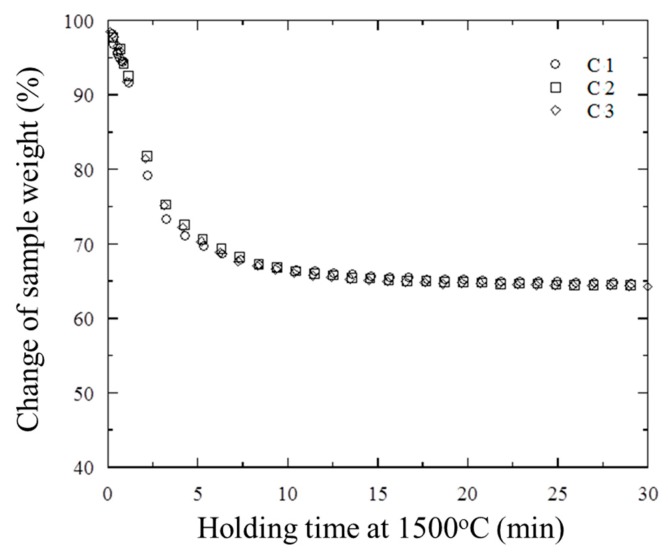
Typical changes of the weight of briquette samples over time during the TGR experiments.

To evaluate the repeatability of data obtained during the TGR experiments, three trials for each type of briquette were done using the same experimental conditions. Typical changes of the sample weight during the TGR experiments are shown in Figure 3 for experiments C1, C2 and C3 with Type C briquette samples. It can be seen that deviations in the results obtained in the C1, C2 and C3 experiments varied from 0.3% up to 2.5% during a holding time of 30 min. A similar tendency was observed in the experiments with Type A briquette samples. Therefore, it was concluded that the obtained results demonstrated a very good repeatability of the given TGR experimental conditions for both types of industrial briquettes. 

The fast decrease of the sample weight during the first 5 to 7 min of the holding time of the samples in the hot zone can be explained by the intensive reductions of Fe, Ni and Cr oxides by carbon in the anthracite and pet-coke in the briquettes at 1500 °C. This occurs due to the following reactions (Equations (2)–(3)):

Fe_2_O_3_ + 3C = 2Fe + 3CO (g) (2)

NiO + C = Ni + CO (g)(3)

Cr_2_O_3_ + 3C = 2Cr + 3CO (g) (4)

**Figure 4:**
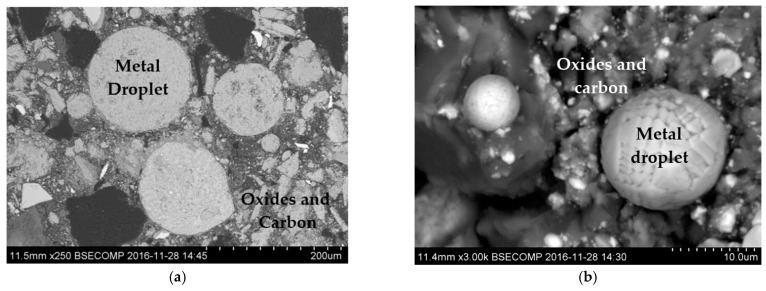
SEM images of different metal droplets (**a**) and (**b**) observed in the slag after completion of the TGR experiments.

This reduction of the Fe, Ni and Cr oxides leads to the formation of metal droplets of different sizes. Typical SEM images of metal droplets observed in the slag after the TGR experiments are shown in Figure 4. It was found that the observed diameter of the metal droplets, D_(MD)_, varied widely in the range of 2 µm up to ~2.0 mm.

**Figure 5:**
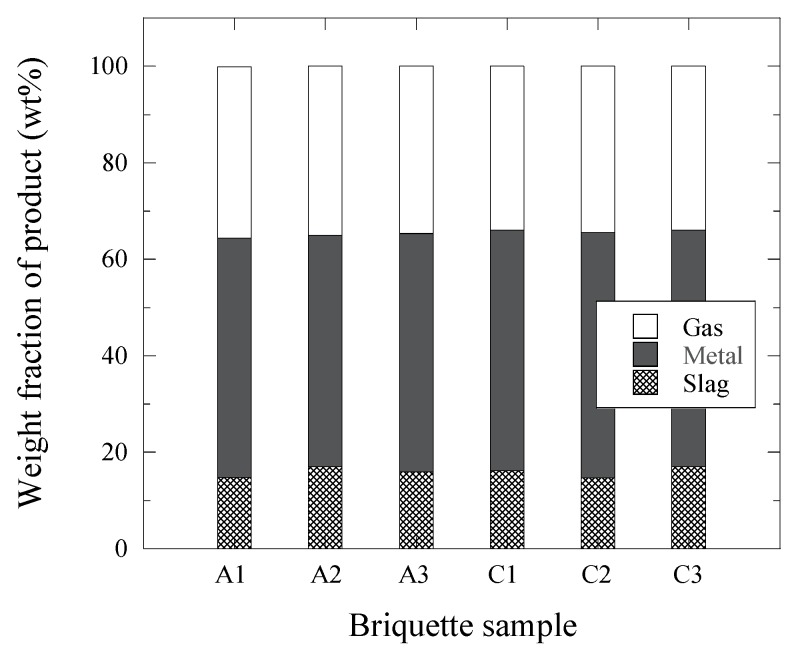
Amounts of gas, metal and slag formed from different briquettes during the TGR experiments at 1500 °C.

By assuming that only the inner components of the briquettes were involved in these reactions and that the formed gas is CO, the decrease of the sample weight during the TGR experiments was found to correspond to the weight of the formed CO gas. Thus, the briquette samples at 1500 °C form a gas (~34–36% from the initial weight of briquette sample), a reduced metal (~48–51%) and a remaining slag (~15–17%). The amounts of gas, metal and slag formed from the briquette sample during the TGR experiments are shown in Figure 5. It can be seen that the amounts of gas, metal and slag obtained during all TGR experiments are very similar for the different briquette samples. For instance, deviations in the weight of the metal part measured after the TGR experiments from the average value for each type of briquette are smaller than 1%. These results once more confirm the repeatability of the TGR experiments for current experimental conditions.

Table 2 and Table 3 show the average compositions of the metal and slag after the completed TGR experiments obtained from the SEM-EDS analysis. It should be noted that the content of Al_2_O_3_ in the slag increases significantly during experimentation due to some erosion of the Al_2_O_3_ crucible. However, the influence of an additional amount of Al_2_O_3_ eroded from the crucible walls on the final composition of slag formed from a briquette was eliminated by using mass balance calculations. As follows from Table 2 and Table 3, the compositions of the obtained metals and slags are typical for the production of stainless steels.

The amount of metal, which was recovered from the briquettes during the TGR experiments, was evaluated by using the mass balance and stoichiometric calculations described in previous work [22,23]. According to the obtained results, the amounts of Cr extracted from the briquettes with anthracite and pet-coke are 93–94% and 93–99%, respectively. The amounts of extracted Fe and Ni are almost 100% for both types of briquettes. Moreover, it should be pointed out that most (84–86%) Fe, Ni and Cr was reduced during the first 5 to 7 min of holding at 1500 °C. Also, the anthracite and pet-coke showed similar good reductant properties during the extraction of these metals from the oxides in the briquettes. A relatively lower extraction amount of Cr (93–99%) in comparison to Fe and Ni (~100%) may be explained by a slower reduction rate of chromium oxides by carbon agents in the briquettes at the given temperature.

**Figure 6:**
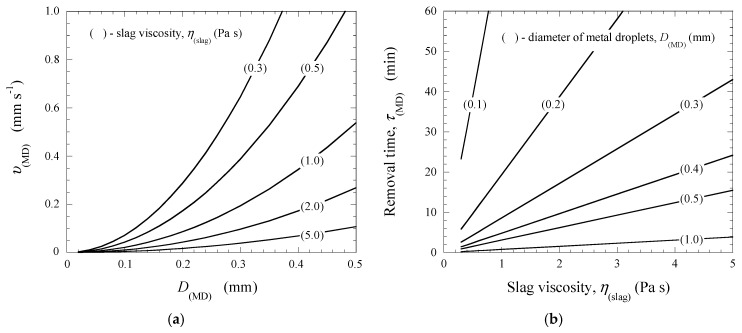
Calculated velocity, υ_(MD)_, and time, τ_(MD)_, for the removal of metal droplets from the liquid slag depending on the size of metal droplets, D_(MD)_ (**a**), and the slag viscosity, η_(slag)_ (**b**).

The metal droplets, which formed from briquettes at high temperatures, can lower from the liquid EAF slag due to significant differences in the densities of the metal droplets (~7150 kg·m^−3^) and slag melt. The values of the removing velocity of the liquid metal droplet, υ(MD), in the unstirred liquid slag were calculated by using Equation (1). However, the density of liquid slag, ρ_(slag)_, and the dynamic viscosity of the liquid EAF slag, η_(slag)_, varied in relatively wide ranges depending on slag composition (basicity of slag, Cr_2_O_3_ content, etc.) and temperature. For instance, the ρ_(slag)_ values for liquid stainless steelmaking slags containing 2–15% Cr_2_O_3_ varied from 3066 to 3410 kg·m^−3^ [15,21]. In this study, a ρ_(slag)_ value of 3200 kg·m^−3^ was used for the evaluation of the sinking velocities of the liquid metal droplets in the liquid slag. The υ_(MD)_ values were calculated for different sizes of metal droplets in low viscosity slags (LVS, η_(LVS)_ = 0.3–1.0 Pa·s) and in high viscosity slags (HVS, η_(HVS)_ = 1.0–5.0 Pa·s) [15,21]. Moreover, the time required for the separation of different metal droplets from the liquid slag, τ_(MD)_, was also estimated for the slag layer in the furnace. The slag thickness was assumed to be 100 mm, which is close to values used in industrial practice. The obtained results are shown in Figure 6.

It was found that all droplets larger than 0.3 mm can be separated completely from LVS slags at around 9 min because the minimum values of υ_(MD)_ for D_(MD)_ = 0.3 mm are almost 0.2 mm·s^−1^. However, the metal droplets with a size of smaller than 0.3 mm can only be partially removed. This is due to the fact that the τ_(MD)_ value varies from 3 to 77 min for D_(MD)_ = 0.29 and 0.1 mm, respectively. In this case, the removal velocities of those metal droplets only reached values of between 0.6 and 0.02 mm·s^−1^, respectively. These calculated data agreed well with the experimental results obtained in this study. Most of the metal droplets, which were observed in the slag samples after the TGR experiments, have a diameter of smaller than 0.3 mm. In the HVS slags (η_(HVS)_ = 1.0–5.0 Pa·s), the metal droplets with D_(MD)_ = 0.5–1.0 mm can be removed completely from the slag during 1 to 16 min depending on the size of the metal droplets and the viscosity of the slags. The separation times τ_(MD)_ for other size ranges of droplets varied from 3 to 43 min for D_(MD)_ = 0.3–0.5 mm and from 9 to 390 min for D_(MD)_ = 0.1–0.3 mm. This means that most droplets in the size range of 0.3 to 0.5 mm and only a small part of the droplets in the size range of 0.1 to 0.3 mm can be removed from the HVS slags during the EAF process. However, it should be noted that foaming EAF slags can have significantly lower densities compared to the slag density used for calculations in this study. This would make it easier to transfer an overwhelming majority of metal droplets from the slag phase to the metal phase. 

The results also showed that the overwhelming majority of the recovered metal (about 93–97%) after the TGR experiments was observed at the bottom of the crucible in the form of a large metal tablet (3.4–3.6 g of metal). Moreover, only 3–8% of the recovered metal was distributed in the form of metal droplets in the slag. Apparently, the small metal droplets can grow faster by merging together to form larger droplets. Thereafter, they can easily be removed from the liquid slag due to an increased sinking velocity. However, it should be noted that intensive stirring of an EAF slag during foaming can significantly change the behavior of the metal droplets recovered from the briquettes as well as their removal from the slag to the steel bath.

During the industrial production of stainless steel in the EAF (85 ton capacity), 1–3 ton of these briquettes can be added to form a foaming slag. In this case, the amounts of Fe, Ni and Cr extracted from the given briquettes reached values of approximately 330, 28 and 66 kg per ton of added briquettes, respectively. This could save a significant amount of expensive ferroalloys and alloys used during the production of stainless steels. In addition, this could contribute to the recycling of waste products from the stainless steel industry as well as improve slag foaming during the EAF process. 

Future trends in the studying of mill-scale based briquettes applied for slag foaming in the EAF steelmaking process could include a wider kinetic study at various temperatures and gas atmospheres in order to research reduction process at different time steps. 

## 4. Conclusions

The recovery of Fe, Ni and Cr from oxides present in briquettes, which can be used for slag foaming in the Electric Arc Furnace (EAF) during stainless steel production, was investigated in laboratory thermo-gravimetric reduction (TGR) experiments. The following conclusions were obtained based on the results obtained from mass balance calculations, thermo-gravimetric experiments and energy-dispersive spectroscopy investigations:

1. The main products, which were obtained during a reduction of Fe_2_O_3_, NiO and Cr_2_O_3_ from rolling mill-scale with anthracite and pet-coke in the given briquettes, are metals (totally 47–50 wt% of Fe, Ni and Cr), gas (33–35 wt%) and slag (15–17 wt%).
2. Up to 95–97% of metal droplets recovered from the briquettes can be removed from the slag in 30 min of the TGR experiments. Only 3–5% of the recovered metal was distributed in the slag in the form of metal droplets having diameters of mostly smaller than 0.3 mm.
3. Almost 100% of Fe and Ni were extracted from the given briquettes at 1500 °C. Moreover, approximately 93–99% of Cr were extracted from these briquettes at the given conditions of the TGR experiments. By using 1 to 3 ton of such briquettes for slag foaming in the EAF, up to 330, 28 and 66 kg of Fe, Ni and Cr, respectively, were recovered from 1 ton of added briquettes.

## Figures and Tables

**Table 1:** Average chemical compositions of different industrial briquettes (in wt%).

Briquette *	C	FeO_x_	NiO	Cr_2_O_3_	SiO_2_	Al_2_O_3_	MgO	CaO	MnO	S
A (anthracite)	21.72	47.56	3.34	11.36	3.77	1.12	0.40	8.56	1.29	0.88
C (pet-coke)	20.16	51.26	3.44	10.81	2.96	1.16	0.35	7.53	1.31	1.01

* Source of carbon in briquette.

**Table 2:** Average compositions (SEM-EDS analysis) of the metals obtained from different briquettes after completion of the TGR experiments (in wt%).

Briquette	Fe	Cr	Ni	C	Al	S
A	71.15	13.93	6.12	7.55	0.09	1.15
C	69.95	14.27	6.01	8.78	0.15	0.84

**Table 3:** Average compositions (SEM-EDS analysis) of the slags obtained from different briquettes after completion of the TGR experiments (in wt%).

Briquette	CaO	SiO_2_	Al_2_O_3_	MnO	MgO	Cr_2_O_3_	FeO	S
A	56.70	17.19	11.77	5.12	2.69	3.96	0.49	2.09
C	57.35	16.52	12.67	4.03	3.94	1.03	0.00	4.46
